# The role of natural products in improving lipid metabolism disorder-induced mitochondrial dysfunction of diabetic kidney disease

**DOI:** 10.3389/fphys.2025.1624077

**Published:** 2025-06-24

**Authors:** Yingping Deng, Han Zhu, Jie Xing, Junwei Gao, Jiamin Duan, Peng Liu, Guanghui Zhong, Xudong Cai

**Affiliations:** ^1^ Department of Nephrology, Ningbo Municipal Hospital of Traditional Chinese Medicine (TCM), Affiliated Hospital of Zhejiang Chinese Medical University, Ningbo, China; ^2^ Graduate School of Jiangxi University of Chinese Medicine, Nanchang, China; ^3^ Xiyuan Hospital, China Academy of Chinese Medical Sciences, Beijing, China

**Keywords:** diabetic kidney disease, lipid metabolism, mitochondria, monomers, Chinese medicine

## Abstract

Diabetic Kidney Disease (DKD) is one of the most common microvascular complications of diabetes mellitus and an important cause of end-stage renal disease, with a complex pathogenesis and a lack of effective treatment. Lipid metabolism disorders play a key role in the progression of DKD, mainly by inducing mitochondrial dysfunction which in turn promotes renal injury. In recent years, natural products have shown great promise in improving lipid metabolism and mitochondrial homeostasis by virtue of their advantages of multi-targeting and low toxicity. In this article, we review the mechanism of mitochondrial dysfunction induced by lipid metabolism disorders in DKD, and the intervention strategies of natural products.

## 1 Introduction

Diabetic Kidney Disease (DKD) is one of the most common microvascular complications of diabetes mellitus and an important cause of end-stage renal disease, with a complex pathogenesis involving a variety of metabolic disorders and cellular damage processes, and limited clinical treatments to effectively stop its progression (Liu et al., 2024). Previous studies have focused on the renal damage caused by hyperglycemia, but in recent years, lipid metabolism disorders have also received extensive attention as an important factor in the progression of DKD (Hou et al., 2024; Zhang et al., 2025).

Mitochondrial dysfunction is particularly pronounced in the context of lipid metabolism disorders, such as impaired fatty acid oxidation, sphingolipid metabolism imbalance, enhanced oxidative stress, and dysregulated mitochondrial homeostasis, which collectively contribute to the process of renal injury and fibrosis (Hou et al., 2024; Narongkiatikhun et al., 2024; Zhou et al., 2023). Natural products show good prospects in regulating lipid metabolism and maintaining mitochondrial homeostasis due to their advantages of multi-targeting and low toxicity, and in-depth study of their molecular mechanisms will help develop new therapeutic strategies to improve the prognosis of DKD patients (Liu et al., 2024; Chen et al., 2023; Chung et al., 2023).

**FIGURE 1:**
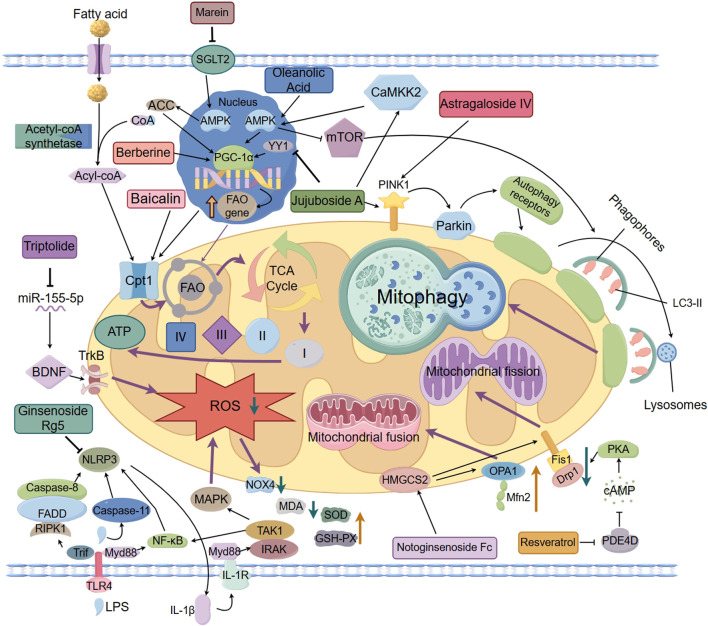
The protective effects of natural products in improving lipid metabolism disorder–induced mitochondrial dysfunction of DKD.

Natural products exert reno-protective effects through multiple pathways, involving a variety of pathological mechanisms such as impaired mitochondrial energy metabolism, enhanced oxidative stress, dysregulated autophagy, and kinetic disorders. The related mechanisms of action are summarized in Table 1 and Figure 1.

**TABLE 1:** Nephroprotective effects of natural products in improving lipid metabolism disorder–induced mitochondrial dysfunction of DKD.

Mechanism	Natural product	Model	Signaling pathways or targets	References
Mitochondrial energy metabolism	Baicalin	db/db mice	CPT1α↑, FAO↑	Hu et al. (2024)
	Berberine	db/db mice and PA induced MPC-5 cells	PGC-1α↑, ROS↓ FAO↑	Qin et al. (2020)
	Oleanolic Acid	HFD/STZ rats	AMPK↑, PGC-1α↑	Pan et al. (2024)
	Jujuboside A	db/db mice	YY1↓, PGC-1α↑	Zha et al. (2024)
	Marein	db/db mice	SGLT2↓,AMPK/ACC/PGC-1α↑	Yin et al. (2025)
Mitochondrial oxidative stress	Ginsenoside Rg5	HFD/STZ mice	NLRP3↓, MAPK↓, ROS↓	Zhu et al. (2020)
	Triptolide	db/db mice	Nrf2↑,GPX4↑ SLC7A11↑,ROS↓	Wang et al. (2024a)
		HFD/STZ mice	miR-155-5p↓, BDNF↑, ROS↓	Gao et al. (2022b)
Mitochondrial autophagy	Jujuboside A	HFD/STZ rats	PINK1/Parkin↑	Zhong et al. (2022)
	Astragaloside IV	db/db mice	Drp1↓, LC-3Ⅱ↑, OPA↑,PINK1↑	Pei et al. (2022)
	Kaempferide	Diet-induced obese mice	TUFM↑, mtROS↑ TFEB↑	Kim et al. (2021)
Mitochondrial dynamics	Resveratrol	db/db mice	PDE4D↓, PKA↑,Drp1(Ser637)↑	Zhu et al. (2023b)
	Notoginsenoside Fc	db/db mice	HMGCS2↓, Drp-1↓, Fis1↓, Mfn2↑	Zhang et al. (2024b)

## 2 Mechanisms of mitochondrial damage induced by disorders of lipid metabolism and intervention of natural products in DKD

The mechanism of mitochondrial dysfunction induced by lipid metabolism disorders includes the following four types: mitochondrial energy metabolism, mitochondrial oxidative stress, mitochondrial autophagy and mitochondrial dynamics. Meanwhile, we have compiled the research progress and therapeutic potential of natural product intervention for mitochondrial damage in DKD.

### 2.1 Mitochondrial energy metabolism

Fatty acid oxidation (FAO) is an important part of mitochondrial energy metabolism, which is mainly carried out in renal tubular epithelial cells. Due to the impermeability of the inner mitochondrial membrane to fatty acyl-CoA, fatty acids rely on the carnitine shuttle system to achieve transmembrane transport. Within this system, the carnitine palmitoyltransferase 1α (CPT1α) isoform of the CPT1 family acts as the first key enzyme, responsible for transferring the fatty acyl group from coenzyme A to carnitine to form acylcarnitine, thereby mediating the entry of fatty acids into the mitochondrial matrix (Console et al., 2020). On this basis, FAO mediates the entry of fatty acids into mitochondria via CPT1α, which is subsequently converted to acetyl-coenzyme A (acetyl-CoA) through a multi-step enzymatic reaction, and enters the tricarboxylic acid (TCA) cycle to generate ATP, and the enhancement of the activity or expression of CPT1 can effectively attenuate renal fibrosis, suggesting that it may be a potential drug target for improving renal function (Lin et al., 2022). Peroxisome proliferator-activated receptor-γ coactivator-1α (PGC-1α), as a transcriptional co-activator, also plays an important role in the regulation of lipid metabolism and mitochondrial function, and it regulates mitochondrial biosynthesis and function by interacting with multiple nuclear receptors and transcription factors (Gudiksen et al., 2025; Zhou et al., 2024; Yu et al., 2023; Chambers and Wingert, 2020). In DKD, due to disordered lipid metabolism, CPT1α and PGC-1α expression is downregulated, resulting in blocked FAO, fatty acids are unable to be oxidized adequately, and accumulate abnormally in renal tubular cells. This lipid accumulation not only directly induced lipotoxicity, but also accelerated apoptosis and renal tubular mesenchymal fibrosis by enhancing the generation of Reactive Oxygen Species (ROS) and the collapse of mitochondrial membrane potential, which further damaged the structure and function of mitochondria (Zhou et al., 2024; Dusabimana et al., 2021).

Baicalin, the main flavonoid constituent of *Scutellaria baicalensis* (a traditional Chinese medicine), exhibits antioxidant, anti-inflammatory, anti-fibrotic, and apoptosis-modulating properties (Li X. et al., 2025). A Recent study has systematically demonstrated, through clinical samples, *in vivo* experiments, and *in vitro* assays, that Baicalin alleviates renal fibrosis in DKD by upregulating CPT1α expression and enhancing FAO. mRNA sequencing revealed significant downregulation of CPT1α in DKD, which was validated by immunohistochemistry in patient renal tissues and in db/db mouse models. *In vitro*, Oil Red O staining and oxygen consumption rate (OCR) assays confirmed that Baicalin effectively improved lipid metabolic disorders and enhanced both mitochondrial respiratory function and FAO capacity (Hu et al., 2024). In addition, a variety of natural products targeting PGC-1α and its related pathways have clear roles in regulating mitochondrial energy metabolism. Berberine is an isoquinoline alkaloid derived from Chinese traditional medicine such as Huanglian, widely used in the fields of hypoglycemia and lipid regulation, anti-inflammation and anti-cancer (Xiong et al., 2022; Li et al., 2023; Zhu L. et al., 2023).A study based on clinical samples and experimental models systematically investigated the potential mechanisms by which lipid metabolic disorders contribute to mitochondrial dysfunction, as well as the therapeutic role of berberine. In the clinical component, plasma samples from patients with DKD were analyzed using gas chromatography–mass spectrometry (GC-MS)-based metabolomics. The results revealed characteristic metabolic alterations, including reduced FAO capacity and abnormal levels of intermediates in the TCA cycle, suggesting that impaired lipid metabolism may be a key contributor to disrupted mitochondrial energy homeostasis. In the experimental component, these findings were further validated using db/db mice and palmitic acid (PA)-treated murine podocyte cell line MPC5. The results demonstrated that berberine exerts protective effects by activating the AMPK/PGC-1α signaling pathway, thereby enhancing FAO, improving mitochondrial function, and effectively alleviating mitochondrial injury and dysfunction caused by lipid metabolic imbalance (Qin et al., 2020). Oleanolic Acid, a pentacyclic triterpenoid widely found in plants such as the traditional Chinese medicine chasteberry, has been demonstrated to possess a variety of biological activities such as anti-inflammatory, immunomodulatory, antioxidant, autophagy-enhancing, and anti-fibrotic activities in renal diseases. In a high-fat diet combined with streptozotocin (HFD/STZ)-induced DKD rat model, Oleanolic Acid can inhibit NF-κB-mediated inflammatory response, regulate mitochondrial energy metabolism, lower blood glucose and lipid levels, and reduce renal lipid deposition by activating the AMPK/PGC-1α pathway (Liu Y. et al., 2022; Pan et al., 2024). Jujuboside A, a triterpenoid saponin isolated from the traditional tranquilizing medicine, Ziziphus jujuba var. spinosa, possesses anti-inflammatory, antioxidant, and calcium homeostasis-regulating effects. In the model of db/db mice, Jujuboside A restores mitochondrial function and inhibits CytC-mediated Caspase9/Caspase3 cascade by down-regulating transcription factor YY1 and enhancing PGC-1α promoter activity to reduce mitochondria-dependent apoptosis and attenuate renal tubular injury associated with DKD (Yang et al., 2025; Zhang W. et al., 2024). Marein, the principal active compound of Coreopsis tinctoria Nutt, exhibits anti-inflammatory and anti-oxidant properties in various diseases, can directly target and inhibit renal tubular SGLT2 expression, activate AMPK/ACC/PGC-1α pathway and reduce renal ectopic lipid deposition, inflammation and fibrosis in the db/db mice model (Zhou et al., 2023; Yin et al., 2025). The above studies provide potential drug targets and therapeutic strategies for intervening in impaired FAO and disturbed energy metabolism in DKD.

### 2.2 Mitochondrial oxidative stress

In DKD, mitochondrial oxidative stress is a key link between lipid metabolism disorders and kidney injury. Studies have shown that abnormal accumulation of free fatty acids (FFA) and lipid intermediate metabolites can induce ROS overproduction, damage mitochondrial structure, and consequently disrupt renal tubular epithelial cell function. Diabetes-related metabolic reprogramming and chronic high-fat dietary intake can further exacerbate oxidative stress and disrupt mitochondrial homeostasis, which in turn drives the continued progression of DKD (Chae et al., 2023; Li and Sheikh-Hamad, 2023). Targeting this mechanism, natural products can alleviate oxidative stress and show good potential for intervention.

Ginsenoside Rg5 (Rg5), a triterpenoid saponin derived from *Panax ginseng*, exhibits multi-target pharmacological activities and has been extensively studied in the fields of anti-inflammation, anti-tumor and neuroprotection (Gao X-F. et al., 2022). In the HFD/STZ-induced DKD mice model, Rg5 intervention significantly improved blood glucose, blood creatinine and uric acid levels, and attenuated glomerular structural damage. In terms of mechanism, Rg5 inhibited the expression of NLRP3 inflammatory vesicle-associated factors (including NLRP3, ASC, and Caspase-1), blocked the activation of inflammatory signals, simultaneously inhibited the phosphorylation of NF-κB and p38 MAPK, and lowered the levels of the pro-inflammatory factors IL-1β and IL-18, which effectively inhibited the inflammatory response in renal tissues. In addition, the levels of oxidative stress markers ROS, NOX4 and TXNIP decreased significantly and the level of MDA was reduced, accompanied by the upregulation of the activities of antioxidant enzymes SOD and GSH-PX in the renal tissues of the mice in the intervention group of Rg5, suggesting that Rg5 plays a synergistic role in anti-inflammatory and antioxidant effects through the regulation of oxidative stress and the cross-regulation of NLRP3/MAPK/NF-κB pathway, thus delaying DKD disease. and thus slowing down the pathologic process of DKD (Zhu et al., 2020).

Triptolide (TP), a triterpenoid extracted from the traditional Chinese medicine *Tripterygium wilfordii Hook. F.*, has anti-inflammatory, antioxidant, and podocytoprotective effects (Li Q. et al., 2025). It was shown that TP intervention significantly improved proteinuria and alleviated glomerular filtration barrier damage caused by the abnormal transformation of the slit diaphragm (SD) to tight junction (TJ) of podocytes in a db/db mouse model, and the mechanism was closely related to the activation of the Nrf2 signaling pathway. TP upregulates the expression of downstream antioxidant factors GPX4, FTH1, and SLC7A11, and inhibits the iron transport protein TFR1, which reduces ROS generation and alleviates mitochondrial oxidative stress and dysfunction (Wang H-Q. et al., 2024). In addition, in the HFD/STZ-induced DKD mouse model, TP intervention effectively reduced fasting blood glucose and urinary protein levels, and ameliorated renal histopathological alterations and ultrastructural abnormalities in podocytes. The mechanism of action of TP involves downregulation of miR-155-5p expression, enhancement of brain-derived neurotrophic factor (BDNF) and podocyte marker protein Nephrin expression, and significant inhibition of inflammatory and oxidative stress factors, such as ROS and IL-1β, to synergistically alleviate the damage and mitochondrial dysfunction of podocytes in a number of pathways. cell injury and mitochondrial dysfunction from multiple pathways (Gao J. et al., 2022). Although natural compounds such as Rg5 and TP have demonstrated potential in mitigating oxidative stress and improving mitochondrial function, current studies predominantly rely on ROS levels and the expression of oxidative stress–related proteins. However, existing evidence lacks direct assessment of mitochondrial function (e.g., respiratory chain activity, membrane potential, and electron microscopy ultrastructure), which limits the mechanistic understanding of their specific targets. Future research should integrate comprehensive functional evaluations to substantiate their mitochondrial protective mechanisms.

### 2.3 Mitochondrial autophagy

Mitochondrial autophagy is the intracellular process of selective degradation of damaged mitochondria, which plays an important role in slowing down the progression of DKD as a key mechanism for maintaining mitochondrial homeostasis. Disturbances in lipid metabolism disrupt the structure and function of the mitochondrial membrane and activate mitochondrial autophagy to remove damaged mitochondria. This process plays an important role in delaying renal injury and disease progression by reducing ROS production, alleviating lipid peroxidation stress, effectively alleviating oxidative stress and blocking apoptotic pathways (Ruan et al., 2024; Shin et al., 2022; Wang et al., 2021).

In DKD, impaired mitochondrial autophagy mediated by the PINK1/Parkin pathway is one of the important mechanisms of mitochondrial dysfunction. Studies have shown that Jujuboside A significantly reduces blood glucose and 24 h urine protein and improves renal tissue structure and function in the HFD/STZ-induced DKD rat model. The mechanism includes activation of CaMKK2-AMPK-p-mTOR signaling axis, upregulation of mitochondrial autophagy key proteins PINK1 and Parkin, enhancement of autophagy activity, and promotion of damaged mitochondrial clearance; at the same time, Jujuboside A also inhibited the expression of NOX4, reduced the production of O_2_- and H_2_O_2_ and enhanced the activities of SOD, CAT, and GPx, and thus alleviated the effects of antioxidant enzymes. Antioxidant enzyme activities, thereby alleviating oxidative stress and mitochondrial respiratory chain disorders, inhibiting the expression of mitochondrial apoptotic proteins such as Bax, CytC, Apaf-1, etc., demonstrating the protective effect of multi-targeted synergistic regulation of mitochondrial function (Zhong et al., 2022). Astragaloside IV (AS-IV) is the main active ingredient of the traditional Chinese medicine *Astragalus membranaceus*, which possesses a variety of pharmacological activities in anti-inflammatory, antioxidant, immune-enhancing and anti-tumor aspects (Zha et al., 2024). AS-IV exhibited significant nephroprotective effects in the db/db mouse model by modulating the mitochondrial quality control network.AS-IV intervention significantly downregulated the expression of mitochondrial cleavage proteins Drp1 and MFF, while up-regulating the expression of the fusion proteins OPA1 and MFN2, as well as mitochondrial autophagy-associated proteins PINK1, Parkin, and LC3-II, which synergistically regulated the mitochondrial This can synergistically regulate mitochondrial cleavage, fusion and autophagy, and maintain the dynamic balance and functional stability of mitochondria (Pei et al., 2022).

Additionally, kaempferide (Kaem), a natural flavonoid from *Kaempferia galanga*, exhibits antiviral, anti-inflammatory, and antioxidant activities such as antiviral, anti-inflammatory, antioxidant, and antifibrotic (Shadman et al., 2025; Zhou et al., 2022; Du et al., 2025). Studies have shown that Kaem induces mitochondrial reactive oxygen species (mtROS) production, promotes lysosomal calcium efflux, and activates the transcription factor EB (transcription factor EB) by directly binding to mitochondrial elongation factor TUFM (mtROS). Transcription factor EB (TFEB) nuclear translocation, which enhances autophagy activity independently of the mTOR pathway. Animal experiments also confirmed that Kaem intervention could promote lipid droplet degradation and alleviate high-fat diet-induced lipid accumulation and metabolic abnormalities, suggesting that it has a good potential to intervene in lipid metabolism remodeling by regulating the mitochondria-autophagy axis (Kim et al., 2021). Although this study did not use a DKD model, it revealed the pathological mechanism of mitochondrial dysfunction induced by lipid metabolism disorders in the context of a high-fat diet, which provides an important reference for further exploring the application of natural products in DKD. In summary, natural products enhance mitochondrial autophagy function and effectively alleviate mitochondrial damage caused by lipid metabolism disorders by targeting the activation of the PINK1/Parkin pathway or regulating the TUFM-TFEB axis.

### 2.4 Mitochondrial dynamics

Mitochondrial dynamics, including the process of mitochondrial fusion and division, is an important mechanism for maintaining the functional integrity of mitochondria and homeostasis of energy metabolism. Recent studies have demonstrated that lipid metabolism disorders in DKD can disrupt mitochondrial dynamic homeostasis by altering mitochondrial membrane lipid composition and the expression of key regulatory proteins (Ge et al., 2020). Li *et al.* reported that carbohydrate-response element-binding protein (ChREBP), a glucose-responsive transcription factor and a central regulator of lipogenesis, not only governs lipid synthesis but also promotes ether phospholipid production through the transcriptional upregulation of glyceronephosphate O-acyltransferase (Gnpat). This process enhances mitochondrial fission and exacerbates mitochondrial morphological abnormalities. Inducible knockdown of ChREBP in podocytes significantly reduced mitochondrial fragmentation and improved the renal phenotype in db/db mice, suggesting that lipid metabolic reprogramming may influence mitochondrial dynamics via modulation of mitochondrial lipid architecture (Li et al., 2023).In addition, abnormal remodeling of cardiolipin—a critical phospholipid of the inner mitochondrial membrane—has been implicated in mitochondrial dysfunction in DKD. Studies have shown that acyl-CoA: lysocardiolipin acyltransferase-1 (ALCAT1) is markedly upregulated in the glomeruli of DKD patients and db/db mice, resulting in the accumulation of oxidized cardiolipin (ox-CL). This accumulation triggers a loss of mitochondrial membrane potential, reduced adenosine triphosphate (ATP) production, and increased reactive oxygen species (ROS) levels. *In vitro* experiments revealed that knockdown of ALCAT1 mitigated high glucose-induced mitochondrial injury, whereas ALCAT1 overexpression exacerbated the pathological changes. Mechanistically, ALCAT1 modulates mitochondrial dynamics through the AMP-activated protein kinase (AMPK) signaling pathway, thereby contributing to excessive mitochondrial fission and impaired mitophagy (Hao et al., 2024).

Lipotoxicity, along with other forms of metabolic stress, constitutes a critical factor contributing to mitochondrial dynamic disequilibrium. Studies have shown that lipotoxicity induces excessive mitochondrial fragmentation and disrupts the balance between fusion and cleavage, which leads to mitochondrial structural damage and dysfunction (Jia et al., 2024; Adebayo et al., 2021; Tanriover et al., 2023), and this imbalance not only affects the normal function of mitochondria, but also exacerbates pathological damage to the kidney (Sun et al., 2025; Li and Susztak, 2025). Excessive cleavage also induces a number of pathological processes such as apoptosis, decreased mitochondrial membrane potential, and impaired respiratory function, which have been demonstrated in a variety of disease models (Liu et al., 2023). Among them, the mitochondrial splitting protein Drp1 and the fusion protein Mfn2 are key factors that regulate mitochondrial dynamics. In this context, natural product intervention has attracted attention as a potential strategy for homeostatic regulation of mitochondrial dynamics. a review by Rahmani et al. indicated that a variety of natural products can effectively alleviate mitochondrial dysfunction triggered by excessive cleavage and play a protective role in multi-organ injury models by inhibiting the expression of Drp1, regulating its phosphorylation status and mitochondrial translocation, and other mechanisms (Rahmani et al., 2023). Studies have further shown that dysregulation of lipid metabolism can induce excessive mitochondrial cleavage through activation of the PDE4D/PKA pathway, which promotes the phosphorylation of Drp1, leading to mitochondrial dysfunction. In this process, Resveratrol, a polyphenolic natural product found in grapes, thujone and other plants, has been shown to play an important protective role in the regulation of mitochondrial dynamics, with multiple pharmacological activities such as antioxidant, anti-inflammatory, anticancer, and improvement of obesity (Ei et al., 2025; Ibars-Serra et al., 2025). In the db/db mice model, Resveratrol inhibits the dephosphorylation of Drp1 at the Ser637 site by activating the PDE4D/PKA signaling axis, thereby blocking mitochondrial cleavage and membrane potential decrease, improving mitochondrial function, and mitigating DKD-associated kidney injury (Zhu X. et al., 2023). Notoginsenoside Fc is a protopanaxadiol-type saponin extracted from the leaves of *Panax notoginseng*, a traditional Chinese medicine, with various pharmacological activities such as antiplatelet aggregation, improvement of vascular endothelial function, anti-inflammatory and antioxidant properties. Research indicates that Notoginsenoside Fc can regulate the HMGCS2 pathway to inhibit the expression of Drp1 and Fis1 in the db/db mice model, enhance mitochondrial fusion mediated by Mfn2, maintain mitochondrial dynamics balance, and downregulate proteins related to proptosis such as TXNIP, NLRP3 and GSDMD-NT, thereby significantly improving mitochondrial damage and alleviating cell proptosis (Shen et al., 2024; Zhang Y. et al., 2024). In summary, abnormal lipid metabolism exacerbates mitochondrial dysfunction in DKD by altering mitochondrial membrane lipid composition, expression of key regulatory factors and related signaling pathways. Targeting core nodes such as ChREBP, ALCAT1 and Drp1, combined with natural products to intervene in the cleavage-fusion imbalance, is expected to provide a new strategy to slow down the progression of DKD.

## 3 Conclusion and perspectives

Mitochondrial dysfunction driven by lipid metabolic disorders constitutes a key mechanism in DKD pathogenesis. By virtue of multi-target regulation and good safety, natural products have shown broad application prospects in improving lipid metabolism abnormalities and restoring mitochondrial homeostasis. In this article, we reviewed a variety of representative active natural products, including baicalin, berberine, oleanolic acid, jujuboside A, marein, Rg5, TP, AS-IV, Kaem, resveratrol and notoginsenoside Fc. These natural products exert reno-protective effects through multiple pathways, effectively intervening in DKD-related mitochondrial damage and dysfunction. Their mechanisms of action include: activating the AMPK/PGC-1α pathway to enhance FAO; inhibiting the overproduction of mitochondrial ROS to alleviate oxidative stress; regulating the PINK1/Parkin pathway to promote mitochondrial autophagy; and targeting the Drp1/Mfn2 pathway to maintain the dynamic balance of mitochondria. Although the therapeutic potential of natural products in DKD has received widespread attention, their safety concerns cannot be ignored. For example, TP possesses pharmacological activities such as anti-inflammatory, antioxidant and immunomodulatory activities, which can ameliorate the pathological damage in DKD. However, several studies have shown that this natural product exhibits significant dose-dependent toxicity under high dose conditions involving multiple systems such as the liver, kidney, intestinal tract and reproductive organs (Wang L. et al., 2024; Liu Y-T. et al., 2022; Wang et al., 2023). Therefore, in addition to in-depth research on the pharmacodynamic mechanisms and targets of the natural product, its toxicological evaluation and dose-effect relationship studies should be strengthened to clarify the safe dose range, so as to provide a reliable guarantee for the clinical transformation of the natural product.

Although some progress has been made in basic research on natural products for the treatment of DKD, most of them focus on a single target or signaling pathway and lack multi-omics integration from gene, transcription, protein to metabolism level. Clinical evidence is still limited, and most of the existing studies have focused on the intervention of traditional Chinese medicine (TCM) compounding, which involves complex components and is difficult to systematically reveal its mechanism of action. Although previous studies based on clinical samples from patients with DKD have employed techniques such as immunohistochemistry (IHC), Western blotting, metabolomics (including lipidomics), and transmission electron microscopy (TEM) to reveal a close association between impaired FAO, TCA cycle disruption, mitochondrial membrane lipid remodeling, and mitochondrial dysfunction (Hu et al., 2024; Qin et al., 2020; Hao et al., 2024), these investigations have primarily focused on disease mechanisms. To date, there is a lack of clinical validation regarding the effects of natural products, particularly single-compound herbal constituents. Current evidence largely stems from preclinical studies, and prospective randomized controlled clinical trials are still lacking, limiting the comprehensive evaluation of their therapeutic efficacy and safety. Future studies should incorporate single-cell RNA sequencing (scRNA-seq) to delineate the cell-type–specific responses within glomeruli and proximal tubules under conditions of lipid metabolic imbalance and mitochondrial dysfunction. The application of spatial transcriptomics will enable the spatial co-localization of metabolic activity and the tissue microenvironment. In addition, the use of multi-platform metabolomics technologies (e.g., ^1H-NMR and LC-MS) can facilitate the dynamic tracking of key metabolic pathways such as FAO and the TCA cycle. In terms of disease modeling, human kidney organoids derived from induced pluripotent stem cells (iPSCs) have been successfully applied to recapitulate podocyte development under specific genetic backgrounds, providing a novel platform for investigating lipid metabolism–induced cellular injury in DKD (Wang G. et al., 2024). Moving forward, there is an urgent need to design and implement high-quality clinical trials on the basis of mechanistic research, in order to effectively bridge experimental evidence and clinical application of natural products. Through the comprehensive integration of multi-omics data and clinical sample validation, the regulatory mechanisms by which natural products modulate the “lipid metabolism–mitochondrial function axis” are expected to be systematically elucidated, thereby offering precise therapeutic targets and advancing translational strategies for DKD intervention.
